# Satisfaction Domains Differ between the Patient and Their Family in Adult Intensive Care Units

**DOI:** 10.1155/2016/9025643

**Published:** 2016-12-01

**Authors:** Amartya Mukhopadhyay, Ge Song, Pei Zhen Sim, Kit Cheng Ting, Jeffrey Kwang Sui Yoo, Qing Li Wang, Raudhah Binte Haji Mohamad Mascuri, Venetia Hui Ling Ong, Jason Phua, Yanika Kowitlawakul

**Affiliations:** ^1^Medical Intensive Care Unit, Division of Respiratory and Critical Care Medicine, National University Hospital, National University Health System, Singapore; ^2^National University Hospital, National University Health System, Singapore; ^3^Division of Respiratory and Critical Care Medicine, National University Hospital, National University Health System, Singapore; ^4^Alice Lee Centre for Nursing Studies, Yong Loo Lin School of Medicine & National University Health System, Singapore

## Abstract

*Background.* Patients' and family's satisfaction data from the Asian intensive care units (ICUs) is lacking.* Objective.* Domains between patient and family satisfaction and contribution of each domain to the general satisfaction were studied.* Method.* Over 3 months, adult patients across 4 ICUs staying for more than 48 hours with abbreviated mental test score of 7 or above and able to understand English and immediate family members were surveyed by separate validated satisfaction questionnaires.* Results.* Two hundred patients and 194 families were included in the final analysis. Significant difference in the satisfaction scores was observed between the ICUs. Patients were most and least satisfied in the communication (4.2 out of 5) and decision-making (2.9 out of 5) domains, respectively. Families were most and least satisfied in the relationship with doctors (3.9 out of 5) and family's involvement domains (3.3 out of 5), respectively. Domains contributing most to the general satisfaction were the illness management domain for patients (*β* coefficient = 0.44) and characteristics of doctors and nurses domain for family (*β* coefficient = 0.45).* Discussion.* In an Asian ICU community, patients and families differ in their expectations and valuations of health care processes. Health care providers have difficult tasks in attending to these different domains.

## 1. Introduction

Over the last three decades, surveys on health service delivery and patient satisfaction have increasingly played important roles as quality indicators to improve and evaluate the outcomes of care provided by health care organizations [1]. Today, with an increased cost of health care, rapid advancement in medical science and technology, rising medicolegal implications, and the impact of knowledge globalization, patients and their families are more critical about health care delivery and medical excellence [2]. As a result, hospitals are committed to proper accountability and efficient use of public money, and patient and family satisfaction continue to be one of the widely used measures in quality assurance [3–5]. The American Academy of Nursing Panel recommends patient satisfaction to be included in the measurement of performance outcome of health care services [6] and the Society of Critical Care Medicine has provided guidelines to support families in intensive care units (ICUs) [7].

Critically ill patients consider physicians' care as the most important component of disease management compared to the other health care services provided to them [8]; therefore, physician-related factors can promote higher levels of satisfaction [9]. Many patients in ICUs are critically unwell, sedated, paralyzed, and unable to communicate. As such, the viewpoints of families become highly relevant [10]. On the other hand, current evidence based guidelines suggest applying minimum sedation, daily awakening, and early mobilization in ICU [11, 12]. Hence vast majority of patients in the contemporary ICUs are awake and able to interact with health care professionals. Often, patients deteriorate rapidly before admission to ICUs, leaving much less time for relatives to prepare for these catastrophic events. Therefore, adequate communication and good decision-making are the two predictors of family satisfaction [13].

The goal of ICU care is to assess and treat life-threatening physical diseases while supporting the psychosocial well-being of patients and their families. Regardless of the clinical outcomes, satisfaction of critically ill patients and their families is an important quality indicator. Multiple studies in North America and Europe [14–16] have elucidated factors related to either patients' or families' satisfactions in ICU care. Satisfaction is a balance of expectations and actual care delivered and heavily dependent on societal perception of adequate care. However, previous studies have failed to explain the total variation of patients' experience based only on the domains of the satisfaction surveys done; unexplained variances may be patients' experiences, their health statuses, and expectations from the healthcare by the society at large [17]. In this context, comprehensive data from Asian ICUs is lacking. A clear understanding of the current practice is essential to any systematic attempt to improve satisfaction in ICUs. Hence, this study was done for collection of baseline data to examine factors which have impacts on patients' and families' satisfaction of the quality of care in ICUs and to compare the contributing domains for patients and families.

## 2. Materials and Methods

### 2.1. Design

This was a cross-sectional descriptive study. Self-reported questionnaires were used to assess patients' and family's satisfaction levels of the health care services delivered in the ICUs.

### 2.2. Setting and Sample

The study was conducted in a 1,000-bed tertiary academic medical center in Singapore involving four adult ICUs: Medical ICU (MICU), Surgical ICU (SICU), Cardiothoracic ICU (CTICU), and Coronary Care Unit (CCU). The inclusion criteria for patients were (1) at least 21 years old, (2) alert and oriented with a minimum abbreviated mental test score of 7 out of 10 to indicate whether they were cognitively sound [18], (3) stayed in the ICU for more than 48 hours, and (4) able to understand English. The inclusion criteria for family participants were (1) at least 21 years old, (2) an immediate family member of the patient who visited the patient at least once during their stay in the ICU, and (3) able to understand English.

### 2.3. Instruments

Canadian Health Care Evaluation Project (CANHELP) questionnaires were used with permission from the authors to assess patients' and families' satisfaction of health care service in ICUs [19].

The CANHELP questionnaire for patients consists of two sections. Section one is demographic data that consists of 10 items and section two consists of 38 items using a 5-point Likert scale (1 = not at all satisfied to 5 = completely satisfied). Item 1 in section two measures general satisfaction with the quality of care perceived by the patients. Subsequent items 2 to 38 are divided into six domains, namely, relationship with doctors, illness management, communication, decision-making, role of the family, and well-being.

The CANHELP questionnaires for families also consist of two sections. Section one is demographic data (10 items) and section two consists of 40 items using a 5-point Likert scale (1 = not at all satisfied to 5 = completely satisfied). Item 1 in section two measures families' general satisfaction with the quality of care patients had received. Item 2 measures families' general satisfaction of how they were treated by health care professionals in the ICU. Items 3 to 40 are divided into 6 domains: relationship with doctors, characteristics of doctors and nurses, illness management, communication and decision-making, families' involvement, and well-being. There are 6 domains which are common in both patient's and family's satisfaction questionnaires.

The CANHELP questionnaires were validated in large, heterogeneous groups of patients across diverse settings and families [20, 21]. In these studies, the overall internal consistencies (Cronbach's alpha) of CANHELP for patient and family were 0.96 and 0.97, respectively. The internal consistencies of the subscales ranged from 0.81 to 0.93 for patients and from 0.80 to 0.92 for families. Therefore, the internal consistencies for each subscale and total scale were acceptable for the current study.

### 2.4. Data Collection

Ethical approval from the domain specific review board of the National Healthcare Group was obtained before data collection. We used admission details to trace the patients' location and information. All eligible patients and family members were approached by the investigators in person. The investigators informed the participants about the purpose of the study and implied consent was obtained. The investigators were available on site to clarify any inquiries from the participants. The participants returned their answered questionnaires in an envelope directly to the investigators. All patients' and family members' information was kept confidential.

### 2.5. Data Analysis

All answered questionnaires were collated and screened for any errors before entry into the IBM SPSS 20.0 software (IBM Corp., Armonk, New York) for analysis. All of the entered data were cross-checked against the questionnaires to ensure accuracy. Data screening was performed to identify missing data and outliers. Data that had a missing value of more than 5% were excluded. Descriptive statistics such as the percentage, mean, and standard deviation were used to summarize the demographic profiles of the participants. Independent *t*-test was used to compare the mean differences of total satisfaction scores between two groups such as gender (male versus female), marital status (married versus others), race (Chinese versus others), education level (secondary school and below versus tertiary), and employment status (working versus not working). For variables that had more than 2 groups, one-way analysis of variance (one-way ANOVA) with post hoc Bonferroni test was used to compare the mean differences of satisfaction total scores between groups (e.g., four units of ICUs). Multiple linear regression analysis was used to identify the most influential factors of patient and family satisfaction on the quality of care. The assumptions of multiple regression analysis were considered. The multivariate normality and linearity for the study were assessed from normal *p*-*p* plots and scatterplots of regression standardized residual. The multicollinearity was assessed from the tolerance and variance inflation factor (VIF) value. All tests were 2-sided and a *p* value of <0.05 was considered significant.

## 3. Results

**Figure 1:**
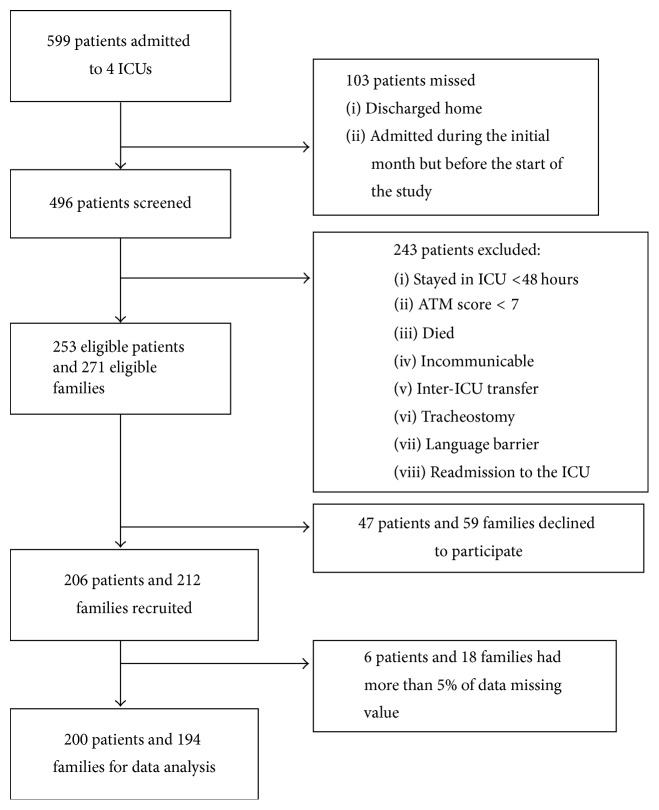
Sample recruitment process. ICU: Intensive Care Unit; AMT: abbreviated mental test.

From November 2012 to January 2013, a total of 599 patients were admitted to the ICUs. According to the inclusion and exclusion criteria, 253 patients and 271 family members were eligible to participate in the study. Two hundred and six patients (81.4 % returned rate) and 212 family members (78.2% returned rate) filled out the questionnaires. After data screening, the final number of participants was 200 patients and 194 family members.  Figure 1 presents the sample recruitment process.

### 3.1. Characteristics of Participants

The majority of the patient participants were male (66.5%) with a mean age of 57.3 (range = 22–91) years. Most patient participants were Chinese (56.5%), married (77.5%), working (55%), living with the family (89.5%), and had attended at least secondary school and below (79.5%). For the family participants, the majority were female (56.2%) with a mean age of 44.9 (range = 21–87) years. Most of the family participants were Chinese (54.1%), married (64.9%), working (66.5%), and had attended secondary school and below (51%). The average ICU length of stay was 3.5 (range = 2–15) days.

### 3.2. Differences between the Participants' Demographic Characteristics on General Satisfactions

Table 1 shows the differences among the demographic characteristics of the participants (both patients and family members) on general satisfactions. There were statistically significant differences in mean general satisfaction scores among the four ICUs for both patients and families (both *p* < 0.001). Patient and family participants in the CTICU had the highest mean satisfaction score (mean ± SD, 4.1 ± 0.4 and 3.8 ± 0.3, resp.), and patients and family members in the SICU had the lowest mean score (mean ± SD, 3.4 ± 0.8 and 3.1 ± 0.7, resp.). According to the post hoc Bonferroni test (Table 2), there were statistically significant differences in general satisfaction score of patients between SICU and MICU (*p* = 0.02), SICU and CTICU (*p* < 0.001), and SICU and CCU (*p* < 0.001). However, there were no significant differences in general satisfaction scores between CTICU and MICU (*p* = 0.60) and between CTICU and CCU (*p* = 1.0). For family participants, the post hoc test (Bonferroni) showed that there were significant differences in general satisfaction score between SICU and MICU (*p* = 0.001) and SICU and CTICU (*p* < 0.001), but no significant differences between SICU and CCU (*p* = 0.40) and between CCU and MICU (*p* = 0.89). There were no statistically significant differences in satisfaction score between gender, marital status, race, education, employment status, and living with family or being alone for patient participants. For family participants, there were statistically significant differences of satisfaction score between races (*p* = 0.006) and employment statuses (*p* = 0.003). Family participants who were Chinese had significantly less general satisfactions (mean ± SD, 3.3 ± 0.6) than family participants of other ethnicities such as Malay and Indian (mean ± SD, 3.6 ± 0.5). Also, family participants who were not working had significantly less general satisfactions (mean ± SD, 3.3 ± 0.6) than those participants who were employed (mean ± SD, 3.6 ± 0.5). There were no statistically significant differences in gender, marital status, and education.

Table 3 presents the differences between patients' and family members' general satisfactions in domains scores. Family members were less satisfied than the patients in the general satisfaction scale (3.5 versus 3.9). Patients were most satisfied with communication and least satisfied with decision making. The corresponding domains for the family were characteristics of doctors and nurses and family involvement, respectively.

### 3.3. Influential Factors on Satisfactions of the Quality of Care

We used the score of general satisfaction with the quality of care as the dependent variable. The independent variables for patient participants were the six satisfaction domains (illness management, relationship with doctors, decision-making, well-being of patient, communication, and role of family). For family participants, the independent variables were the same, except that characteristic of doctors and nurses domain was used instead of decision-making domain. The *q*-*q* plot result showed a straight line for normality, and scatterplots showed a rectangle shape with scores in the center and clustering around the zero line. The tolerance values ranged from zero to ten, and VIF values were less than 10 for all variables. This indicated that the normality, linearity, and multicolinearity, as the assumptions of linear regression, were met.  Table 4 shows the multiple regression analysis results of the factors predicting patients' and families' general satisfactions. For patient, the illness management and relationship with doctors domains were significant predictors of satisfactions on the quality of care in ICUs (*β* = 0.44 and 0.31 resp.; *p* < 0.001 for both domains). For the family participants, characteristics of doctors and nurses and relationship with doctors domains were significant predictors of satisfactions on the quality of care in ICUs (*β* = 0.45 and 0.32 resp.; *p* < 0.001 for both domains). The domain content of the questionnaires explained 75% and 73% of the variations for patients' and family's general satisfactions of quality of care in ICUs, respectively.

## 4. Discussion

The mean patient and family satisfaction scores of 3.9 out of 5 (±0.6) and 3.5 out of 5 (±0.6), respectively, reflect the satisfaction levels with health care services in our ICUs. Patients were very satisfied with the domains of communication, relationship with doctors, and illness management. They were least satisfied with the decision-making domain (Table 3). The domain of illness management contributed the most to the general patient satisfaction score (Table 4). Families were very satisfied with the domain of characteristics of doctors and nurses, followed by relationship with doctors, communication, and illness management. They were least satisfied with family involvement.

As effective medical care is increasingly measured according to economic and clinical criteria and managers promote a “customer service-oriented culture” in health care organizations, the inclusion of patients' opinions in the assessments of services has gained greater prominence over the past years. Satisfaction is a difficult concept to define and generally envisages several components, namely, patients' personal preferences, expectations, and the realities of the care received, each component being affected by several factors including patients' previous experiences with the health care system and societal beliefs. ICU experiences by the patient and their families can be positive [22, 23], negative [22, 24], and have lasting effects even after discharge [25]. Communication between health care professionals and patients/family members in an ICU setting is vital. As patients are often unable to speak, both verbal and nonverbal communications are important for each patient's emotional support and subsequent satisfaction [26, 27]. Nurses are present at patients' bedsides and therefore play a crucial role [28]. Interestingly, although our ICUs scored high on communication domain, this was a small component contributing to the patients' general satisfaction that valued illness management as the most influential one. On the contrary, the domain of characteristics of doctors and nurses was scored high by the families and was also the most important domain contributing to the general satisfaction of the families. Although patients and their family members were not exactly congruent in this study, this unique finding calls for further research in this field. Previous experiences and societal perceptions of good healthcare modify patients' current expectations of the services offered to them. This concept is difficult to measure and definitive studies are lacking. Bleich et al. [17] had shown that only 10.4% of the variation of patients' satisfaction in the hospitals of European Union countries was explained by responsive variables (e.g., autonomy, choice, communication, etc.) and they predicted that the large unexplained variation is related to broader societal factors including patients' past experiences. In our study, a large variation (75% for patient and 73% for family) was explained by the domains of the survey. This difference is probably related to the more inclusive questionnaire used in the current study. Singapore is one of the most efficient healthcare systems in the world [29, 30] and the population's impression of public healthcare institutions is generally high. It is therefore also possible that the questionnaire elicited more local institution associated factors related to the satisfaction. The public is already happy about their healthcare system in general, hence explaining the large variance related to the domains in the questionnaire.

Our ICUs score better in the domains of communication, relationship with doctors, and illness management, both for patients and their families (Table 3). Undoubtedly, these are important domains that are very valuable to the patients and their families. However, scores were relatively low in the domains of decision-making and family's involvement. Empowering the patients in their decision-making processes increases their responsibilities towards their health [31] and in turn improves their satisfactions [32]. In emergency and nonelective circumstances, decision-making on patient's behalf relies on their family members in ICUs. Previous studies have also shown that families may not be satisfied with their involvement in the decision-making [33]; however, in a study involving 78 ICUs in France, the desire to share was expressed by 47% of the family members but only 15% of the family members actually shared the decision-making process [34]. Given that many immediate family members in ICUs suffer from extreme stress and anxiety, which may cloud their decision-making [35], the above results are not surprising. It is to be noted that the agreement between a surrogate's and a patient's decision is poor [36, 37] and intensive care health care professionals have the difficult task of balancing these differences in opinions. Nevertheless, we believe that our ICUs can improve in families'/patients' involvement in decision-making through quality improvement.

Our study has several strengths. We included all adult ICUs serving varied case mixes and a multiracial patient and family population. Patients and their families were approached with validated questionnaires within a short period of time after their discharge from ICUs and return rates were high. A large variation of the general satisfaction was explained by the domain content of the questionnaire, suggesting appropriate use of the questionnaire tool. Weaknesses of the study include the exclusion of family members whose relatives have died in the ICUs and the loss of a significant number of patients due to early discharges and the inability to contact families. Furthermore, due to the type of questionnaires used, we could only include patients and families who were able to understand English. This may have introduced selection bias in the study population.

## 5. Conclusion

In a multiracial Asian ICU community, patients value illness management as the most important domain of their satisfaction while the characteristics of doctors and nurses were the most important to their families, suggesting different perspectives of patients and their family members. The above two domains are not mutually exclusive, but intensive care health professionals have the difficult task of balancing between the expectations of patients and their families.

## Figures and Tables

**Table 1:** Mean differences among demographical data of patients and family participants.

	Patient (*n* = 200)	Satisfaction score (±SD)	Test and significance	Family (*n* = 194)	Satisfaction score (±SD)	Test and significance
*Location*, *n* (%)						
CTICU	63 (31.5)	4.1 (±0.4)	*F* = 11.9 *p* ^a^ < 0.001^*∗∗*^	50 (25.8)	3.8 (±0.3)	*F* = 13.5 *p* ^a^ < 0.001^*∗∗*^
CCU	61 (30.5)	3.9 (±0.4)		28 (14.4)	3.4 (±0.6)	
MICU	43 (21.5)	3.8 (±0.8)		72 (37.1)	3.5 (±0.5)	
SICU	33 (16.5)	3.4 (±0.8)		44 (22.7)	3.1 (±0.7)	
*Gender*, *n* (%)						
Male	133 (66.5)	3.9 (±0.6)	*t* = 0.51 *p* ^*t*^ = 0.607	85 (43.8)	3.6 (±0.5)	*t* = 1.93 *p* = 0.051
Female	67 (33.5)	3.8 (±0.7)		109 (56.2)	3.4 (±0.6)	
*Age, years*						
Mean (SD)	57.3 (±14.9)	3.8 (±0.6)		44.9 (±15.6)	3.5 (±0.5)	
Range	22–91			21–87		
*Marital status*, *n* (%)						
Married	155 (77.5)	3.9 (±0.6)	*t* = −0.01 *p* ^*t*^ = 0.961	126 (64.9)	3.5 (±0.6)	*t* = −1.30 *p* ^*t*^ = 0.195
Others	45 (22.5)	3.9 (±0.6)		68 (35.1)	3.6 (±0.4)	
*Race*, *n* (%)						
Chinese	113 (56.5)	3.8 (±0.7)	*t* = −0.12 *p* ^*t*^ = 0.903	105 (54.1)	3.3 (±0.6)	*t* = −2.79 *p* ^*t*^ = 0.006^*∗*^
Others	87 (43.5)	3.9 (±0.5)		89 (45.9)	3.6 (±0.5)	
*Education*, *n* (%)						
Secondary school and below	159 (79.5)	3.9 (±0.6)	*t* = 0.95 *p* ^*t*^ = 0.342	99 (51)	3.5 (±0.6)	*t* = −0.73 *p* ^*t*^ = 0.464
Tertiary	41 (20.5)	3.8 (±0.7)		95 (49)	3.5 (±0.5)	
*Employment status*, *n* (%)						
Working	110 (55)	3.9 (±0.6)	*t* = −0.19 *p* ^*t*^ = 0.848	129 (66.5)	3.6 (±0.5)	*t* = −2.98 *p* ^*t*^ = 0.003^*∗*^
Not working	90 (45)	3.8 (±0.7)		65 (33.5)	3.3 (±0.6)	
*Living with*, *n* (%)						
Families	179 (89.5)	3.9 (±0.6)	*t* = −0.16 *p* ^*t*^ = 0.874	N.A.		
Alone	21 (10.5)	3.8 (±0.5)				
*LOS, days*						
Mean (±SD)	3.5 (±2.0)	3.8 (±0.6)		N.A.	N.A.	N.A.
Range	2–15					

CTICU: cardiothoracic intensive care unit; CCU: coronary intensive care unit; MICU: medical intensive care unit; SICU: surgical intensive care unit; SD: standard deviation; and LOS: length of stay.

^a^One-way analysis of variance for more than two groups.

^*t*^Independent *t*-test for two groups.

^*∗∗*^Significant level < 0.01.

^*∗*^Significant level < 0.05.

Interpretation of the mean score: 1 = not at all satisfied, 2 = not very satisfied, 3 = somewhat satisfied, 4 = very satisfied, and 5 = completely satisfied.

**Table 2:** Mean differences among four ICUs (post hoc Bonferroni test).

ICU		Patient (*n* = 200)	Significant level (*p* value)	Family (*n* = 194)	Significant level (*p* value)
A	B	Mean difference (A − B)		Mean difference (A − B)	
MICU	SICU	0.40	0.02^*∗*^	0.39	0.001^*∗∗*^
	CTICU	−0.30	0.06	−0.26	0.04^*∗*^
	CCU	−0.20	0.51	0.17	0.89

SICU	MICU	−0.40	0.02^*∗*^	−0.39	0.001^*∗∗*^
	CTICU	−0.70	<0.001^*∗∗*^	−0.66	<0.001^*∗∗*^
	CCU	−0.60	<0.001^*∗∗*^	−0.23	0.40

CTICU	MICU	0.30	0.06	0.26	0.04^*∗*^
	SICU	0.70	<0.001^*∗∗*^	0.66	<0.001^*∗∗*^
	CCU	0.10	1.0	0.43	0.003^*∗∗*^

CCU	MICU	0.20	0.51	−0.17	0.89
	SICU	0.60	<0.001^*∗∗*^	0.23	0.40
	CTICU	−0.10	1.0	−0.43	0.003^*∗∗*^

^*∗∗*^Significant level < 0.01.

^*∗*^Significant level < 0.05.

**Table 3:** Differences between patients' and families' general satisfactions in the domain scores.

Domains	Patient (*n* = 200)	Family (*n* = 194)
	Mean (±SD)	Mean (±SD)
General satisfaction	3.9 (±0.6)	3.5 (±0.6)
Communication	4.2 (±0.7)	3.8 (±0.8)
Relationship with doctors	4.1 (±0.8)	3.9 (±0.7)
Illness management	4.1 (±0.6)	3.8 (±0.6)
Well-being of patient/family	3.7 (±1.0)	3.5 (±0.9)
Role of family/family involvement	3.5 (±1.1)	3.3 (±1.0)
Decision-making	2.9 (±1.2)	N.A.
Characteristics of doctors and nurses	N.A.	4.0 (±0.6)

**Table 4:** Influential factors for patients' and families' satisfactions on quality of care in ICUs.

Independent variables (domains)	Patient (*n* = 200)				Family (*n* = 194)			
	Beta coefficients	*p* value	Tol	VIF	Beta coefficients	*p* value	Tol	VIF
Illness management	0.44	<0.001^*∗∗*^	0.37	2.69	0.11	0.155	0.46	2.19
Relationship with doctors	0.31	<0.001^*∗∗*^	0.48	2.08	0.32	<0.001^*∗∗*^	0.46	2.12
Decision-making	−0.10	0.068	0.77	1.29	N.A.			
Well-being of patient/family	0.12	0.102	0.41	2.46	−0.08	0.219	0.68	1.48
Communication	0.02	0.853	0.67	2.72	−0.03	0.673	0.56	1.79
Role of family/family involvement	−0.02	0.819	0.54	1.85	−0.01	0.869	0.54	1.84
Characteristics of doctors and nurses	N.A.				0.45	<0.001^*∗∗*^	0.39	2.56
*R* ^2^ = 0.75 (patient)					*R* ^2^ = 0.73 (family)			

Tol: tolerance value; VIF: variance inflation factor.

^*∗∗*^Significant at *p* < 0.01.
